# Does it matter that standard preparedness indices did not predict COVID-19 outcomes?

**DOI:** 10.1186/s12992-023-00973-2

**Published:** 2023-09-23

**Authors:** Michael A. Stoto, Christopher D. Nelson, John D. Kraemer

**Affiliations:** 1 Department of Health Management and Policy, School of Health, Georgetown University, Washington DC, USA; 2 RAND Corporation and, RAND Pardee Graduate School, Santa Monica, CA USA

**Keywords:** Public health preparedness, Preparedness measures, Joint External Evaluation, Global Health Security Index, COVID-19 outcomes

## Abstract

A number of scientific publications and commentaries have suggested that standard preparedness indices such as the Global Health Security Index (GHSI) and Joint External Evaluation (JEE) scores did not predict COVID-19 outcomes. To some, the failure of these metrics to be predictive demonstrates the need for a fundamental reassessment which better aligns preparedness measurement with operational capacities in real-world stress situations, including the points at which coordination structures and decision-making may fail. There are, however, several reasons why these instruments should not be so easily rejected as preparedness measures.

From a methodological point of view, these studies use relatively simple outcome measures, mostly based on cumulative numbers of cases and deaths at a fixed point of time. A country’s “success” in dealing with the pandemic is highly multidimensional – both in the health outcomes and type and timing of interventions and policies – is too complex to represent with a single number. In addition, the comparability of mortality data over time and among jurisdictions is questionable due to highly variable completeness and representativeness. Furthermore, the analyses use a cross-sectional design, which is poorly suited for evaluating the impact of interventions, especially for COVID-19.

Conceptually, a major reason that current preparedness measures fail to predict pandemic outcomes is that they do not adequately capture variations in the presence of effective political leadership needed to activate and implement existing system, instill confidence in the government’s response; or background levels of interpersonal trust and trust in government institutions and country ability needed to mount fast and adaptable responses. These factors are crucial; capacity alone is insufficient if that capacity is not effectively leveraged. However, preparedness metrics are intended to identify gaps that countries must fill. As important as effective political leadership and trust in institutions, countries cannot be held accountable to one another for having good political leadership or trust in institutions. Therefore, JEE scores, the GHSI, and similar metrics can be useful tools for identifying critical gaps in capacities and capabilities that are necessary but not sufficient for an effective pandemic response.

## Background

Since the start of the pandemic, a number of scientific publications and commentaries have suggested that scores based on the WHO’s States Party Self-Assessment Annual Reporting tool (SPAR) and the Joint External Evaluation (JEE) tool and the Global Health Security Index (GHSI) did not predict COVID-19 outcomes [1–6]. Citing such results, the Global Preparedness Monitoring Board, in its 2020 report, notes that “The ultimate test of preparedness is mounting an effective response,” suggesting that “our understanding of pandemic preparedness has been inadequate” [7]. To the Independent Panel for Pandemic Preparedness and Response, “the failure of these metrics to be predictive demonstrates the need for a fundamental reassessment which better aligns preparedness measurement with operational capacities in real-world stress situations, including the points at which coordination structures and decision-making may fail” [8].

## Main text

But do the analyses comparing mortality to SPAR and JEE scores and the GHSI really prove that these measures are not valid measures of a country’s preparedness [9, 10]? Predictive validity, the degree to which a measure statistically predicts desirable outcomes, is a common way to assess performance measures. For example, measures of a hospital’s adherence to infection control protocols should be associated with lower surgical mortality rates. Nevertheless, there are several reasons that these instruments should not be so easily rejected as preparedness measures.

First, as Stoto and colleagues [11] note, the comparability of such data (which come from the Johns Hopkins University COVID-19 Dashboard, Worldometer, and similar sources) over time and among jurisdictions is questionable due to highly variable completeness and representativeness [12, 13]. Indeed, countries that have stronger public health systems—and thus higher scores for surveillance in particular and preparedness overall— may be more likely to count COVID-19 cases and deaths completely. This would create a correlation in the wrong direction.

Second, these studies use relatively simple outcome measures, mostly based on cumulative numbers of cases and deaths at the country level and at a fixed point of time. However, a country’s “success” in dealing with the pandemic is highly multidimensional – both in the health outcomes and type and timing of interventions and policies. Performance as measure by cumulative numbers of cases or deaths early in the pandemic might be contradicted by performance in later stages (e.g. when the vaccine became available). Total cases and deaths also do not reflect differences within countries by socio-economic groups, geography, or other factors. In addition, limiting social and economic disruption is an important policy aim that is not addressed by case/death counts. In other words, the impact of capabilities that were highly effective for some groups at a specific time might not be observed in cumulative mortality figures.

Third, the analyses to assess predictive validity use cross-sectional designs with outcome data aggregated over multiple epidemic phases, which is poorly suited for evaluating the impact of interventions, especially for COVID-19 policies, where part of the challenge was to adjust policies to the emergence of new variables and new socio-economic impacts over the long course of the pandemic [11, 14]. Cross-sectional studies are even more problematical where, as in the case of COVID-19, both outcomes and interventions are highly multidimensional, as discussed in the previous paragraph.

Beyond questions of data quality and study design, there is a serious conceptual issue: a major reason that current preparedness measures fail to predict pandemic outcomes is that they do not adequately capture variations in the presence of effective political leadership needed to activate and implement existing system, instill confidence in the government’s response; or background levels of interpersonal trust and trust in government institutions and country ability needed to mount fast and adaptable responses. As Bell, Fukayama, and others have noted, these factors are crucial; capacity alone is insufficient if that capacity isn’t effectively leveraged [15, 16]. These factors might be labeled “social capital, and represent the difference between preparedness and resilience [17].

Ledesma and colleagues [18] recently analyzed the relationship between GHSI scores and COVID-19 outcomes and found a quite different result than the others cited in this section: higher GHSI scores were associated with lower COVID-19 deaths18. Part of the reason for the difference is that these authors avoided problems of undercounting by focusing on excess mortality estimates. They also adjusted for population age and looked at mortality over two years (2020 and 2021) rather than a limited window. Ledesma and colleagues further analyzed the results in a multivariate regression exploring the impact of the six components of the GHSI. Controlling for the other components, they found that the “risk environment score” had a stronger relationship to COVID-19 mortality than any of the others. This score includes government effectiveness, public confidence in governance, trust in medical and health advice, and related factors. Thus, it differs conceptually from the other five components, which are more traditional preprepared measures. This suggests that the GHSI is a good measure of resilience, measuring both preparedness and social capital.

## Conclusions

As always in assessing measurement systems, the purpose is critical. If the goal is, as suggested by the Independent Panel, to identify “the points at which coordination structures and decision-making may fail” [8], analyses of summary metrics can be useful.

Preparedness metrics, however, were not intended to predict outcomes. Rather, the WHO’s SPAR tool was developed to hold countries accountable for fulfilling their obligations under the International Health Regulations [19]. Other measurement systems, such as the JEE tool, are intended to identify gaps in preparedness systems and to allow countries to engage with donors and partners such as UN agencies, local and international nongovernmental organizations to target resources effectively [10]. In other words, it is about what countries do to enhance preparedness capacities, not the outcomes they achieve. For these purposes, the question is not whether the SPAR, JEE, the GHSI and similar metrics predict overall COVID-19 outcomes, but rather whether they identify gaps in preparedness capacities and capabilities that are necessary, but not sufficient, to guarantee good outcomes. As important as effective political leadership and trust in institutions are, countries cannot hold one another accountable for having good political leadership or trust in institutions.

It is also important to consider the nature of the systems that we are seeking to measure and improve. Predictive validity is a perfectly reasonable approach in systems where cause-effect relationships are relatively stable and knowable. However, such reliable “if–then” knowledge is harder to come by in highly complex and changing systems, and where the impact of a factor X may be highly conditional on the any number of contextual factors. Prediction would, of course, be very desirable in such situations. Here we are reminded of Berwick’s widely-read essay “The Science of Improvement,” where he argues that for complex social interventions – “whose effectiveness … is sensitive to an array of influences: leadership, changing environments, details of implementation, organizational history, and much more” [20] – it is necessary to understand the complexities through detailed examples of processes and dynamics. We fear that too much focus on predictive validity – while perhaps understandable – may distract us from this task.

## Data Availability

Data sharing is not applicable to this article as no datasets were generated or analyzed during the current study.

## Abbreviations

COVID-19: Coronavirus disease 2019 (the illness caused by the SARS-CoV-2 virus)
GHSI: Global Health Security Index
JEE: Joint External Evaluation tool
SPAR: States Party Self-Assessment Annual Reporting tool
